# Development of Receptor-Integrated Magnetically Labeled Liposomes for Investigating SARS-CoV-2 Fusion Interactions

**DOI:** 10.1021/acs.analchem.4c05966

**Published:** 2025-02-10

**Authors:** Tuhina Banerjee, Clayton Frazier, Neelima Koti, Paris Yates, Elizabeth Bowie, Megan Liermann, David Johnson, Sharon H Willis, Santimukul Santra

**Affiliations:** 1 Department of Chemistry and Biochemistry, Missouri State University, 901 S. National Avenue, Springfield, Missouri 65897, United States of America; 2 Computational Chemical Biology Core, University of Kansas, 2034 Becker Drive, Lawrence, Kansas 66018, United States of America; 3 Integral Molecular Incorporation, One uCity Square 25 N. 38th Street, Suite 800, Philadelphia, Pennsylvania 19104, United States of America

## Abstract

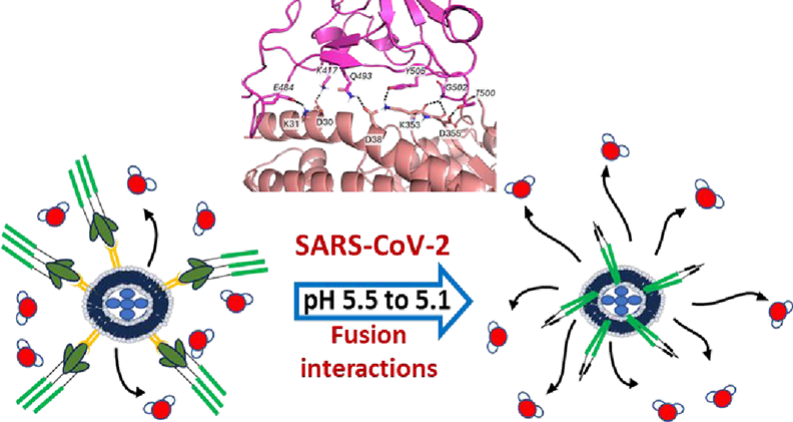

The impacts of highly pathogenic enveloped viruses, such as SARS-CoV-2, have turned scientific inquiry toward the fusion mechanisms responsible for viral pathogenesis and to seek cost-effective and adaptable strategies to mitigate future outbreaks. Current approaches for studying SARS-CoV-2 fusion include computational studies, pan-coronavirus viral inhibitors, and modified peptides and lipopeptides, along with various nanotechniques. Although these methodologies have illuminated the fusion mechanisms, they possess key limitations that prevent their widespread utility in outbreaks, including high financial or instrumental costs, operational proficiency, cytotoxicity, or viral specificity. This work measures changes in spin–spin T_2_ magnetic (transverse) relaxation times using a benchtop NMR instrument and introduces a bioanalytical approach to quickly quantify fusion interactions between the SARS-CoV-2 spike protein and liposome-coated iron oxide nanosensors (LIONs). Additionally, this study modifies the LION platform by appending the angiotensin-converting enzyme (ACE2) receptor, thereby creating LIONs-ACE2 that mimics the ACE2 host cell receptor targeted by SARS-CoV-2. Furthermore, SARS-CoV-2 fusion to other receptors reported to be involved is also examined. Environmental factors impacting fusion, such as calcium ion concentration, cholesterol composition, pH, neutralizing antibodies, and lower temperature, are investigated. Finally, molecular dynamics (MD) simulation studies reveal that the receptor binding domain (RBD) of the spike protein interacts more favorably with ACE2 than the lipid bilayer in the opened conformation, yet the closed conformation of RBD interacts with the bilayer with a similar energy as with ACE2. These findings reveal how the LION platform offers a customizable, fast-acting, inexpensive, and accessible mechanism for examining the fusion process of SARS-CoV-2 and other enveloped viruses.

## Introduction

Major outbreaks of highly pathogenic enveloped viruses within the past decade necessitate a careful examination of the fusion mechanisms enabling viral pathogenesis between host cell membranes and viral envelopes. For example, the SARS-CoV-2 virus, which causes coronavirus disease 2019 (COVID-19), has resulted in nearly 775,000,000 confirmed cases and over 7,000,000 deaths around the world as of April 28, 2024.^1^ Enveloped viruses such as SARS-CoV-2, Ebola, Zika, and influenza utilize viral surface proteins to fuse with host cell membranes and overcome cell entry, one of the greatest barriers to viral propagation.^2^ After fusion, the enveloped virus facilitates the release of its viral genome from the endosome into the cytoplasm of the host cell.^3,4^ SARS-CoV-2 fusion in host cell membranes is facilitated by the S glycoprotein, which is first cleaved into distinct S1 and S2 subunits within infected cells.^5^ Hoffmann et al. and Shang et al. have described how cleavage is achieved by furin or furin-like proteases.^6,7^ The S1 subunit selectively attaches to the ACE2 receptor; next, the S2 subunit affixes the S protein to the virion membrane. Moreover, the S2 subunit includes a fusion peptide that oversees fusion and infection of target cells.^8^ However, after attachment of the S1 subunit to the target cell’s ACE2 receptor, the S2 subunit undergoes an additional cleavage event on its S2’ site via transmembrane protease, serine 2 (TMPRSS2), present at the target cell’s surface.^9−11^ If TMPRSS2 levels at the target cell’s surface are low, then an alternate cell entry mechanism can occur through clathrin-mediated endocytosis, in which cathepsin proteases cleave the S2’ site of the internalized SARS-CoV-2-ACE2 complex.^12−14^ In both routes of entry, S2’ cleavage frees the fusion peptide and generates a fusion pore whereby the viral genome accesses the target cell’s cytoplasm and advances its infection cycle.^5^ Critical opportunities exist for medical interventions within each step of these fusion-mediated cell-entry pathways. Specifically, if binding or fusion is thwarted, then the entire viral life cycle is arrested.

Given the speed and severity with which outbreaks of enveloped viruses have swept across the globe in the past decade, it is imperative to develop a simple, cost-effective, fast-acting, and adaptive technique capable of inhibiting emergent viral fusion pathways. The viral fusion process of enveloped viruses has been illuminated through modern techniques, such as ensemble fusion assays, single viral tracking assay, electron microscopy (EM), cryo-electron tomography (cryo-ET), electrophysiology, single-molecule Forster resonance energy transfer (sm-FRET), and sedimentation equilibrium analytical ultracentrifugation.^15−25^ Specifically, the SARS-CoV-2 fusion mechanism has been carefully elucidated in recent years. The seminal SARS-CoV-2 X-ray crystallographic studies advanced by Shang et al.^26^ and Lan et al.^27^ also demonstrate that the SARS-CoV-2 spike protein is more adaptive and binds more tightly to host cell ACE2 receptors compared to SARS-CoV spike proteins.^28,29^ In addition, advanced computational approaches, immunoassays, synthesized peptides and lipopeptides, pan-coronavirus fusion inhibitors, and various vaccine delivery systems have been explored for their potential to arrest or inhibit SARS-CoV-2 fusion with target cells.^30−36^ Of note, several nanoparticle-based approaches have likewise been studied to examine their effectiveness in preventing SARS-CoV-2 fusion with host cells.^37−43^ Although these techniques have collectively provided important insights into foundational SARS-CoV-2 fusion mechanisms, their concomitant limitations, such as time constraints, high costs, cytotoxicity, expensive instrumentation, viral specificity, and technical requirements, hinder their widespread adoption and diminish their effectiveness in swiftly responding to a panoply of urgent pathogenic outbreaks. These limitations can be markedly exacerbated in developing countries, in which emergent viral outbreaks can have disproportionately severe impacts on healthcare resources and local populations.^44^ Moreover, the current techniques for combating SARS-CoV-2 fusion do not support a single tool capable of quickly, affordably, and adaptively pivoting to inhibit the viral fusion mechanisms of other enveloped viruses. Thus, it is apparent that a customizable modality should be developed to rapidly screen potential fusion inhibitors capable of detecting and responding to a diverse range of pathogenic targets.

**Scheme 1:**
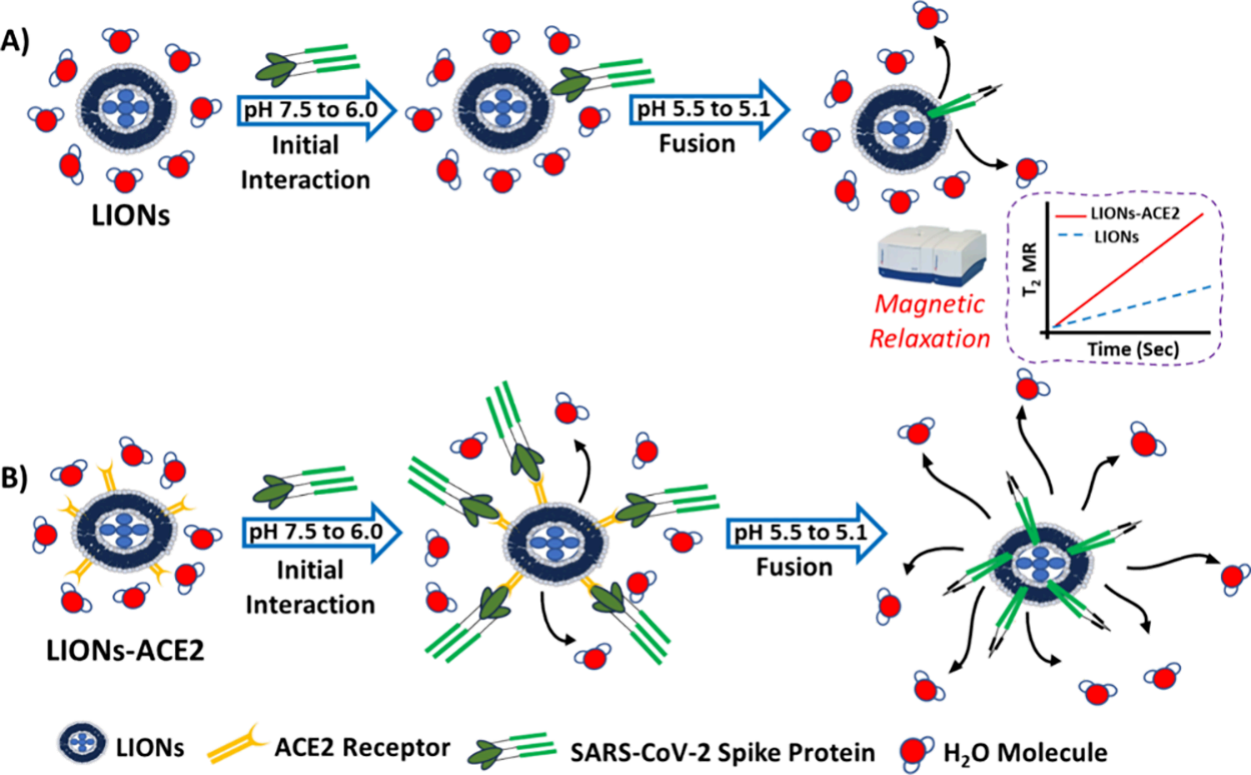
Schematic Representation of the Detection Principle of Transverse Relaxation Assay for Real-Time Quantification of the SARS-CoV-2 Spike Protein and Host Membrane Interactions Using (A) Magnetically Labeled Liposomes (LIONs) and (B) ACE2 Receptor-Integrated Magnetically Labeled Liposomes (LIONs-ACE2)

We have previously reported the successful utilization of a benchtop NMR instrument to evaluate the LION platform capable of demonstrating the viral fusion mechanism of influenza, an enveloped virus using the glycoprotein hemagglutinin to facilitate fusion with the lipid membranes of target cells.^45^ Specifically, as fusion proteins surround the LIONs (Scheme 1A), existing water molecules are displaced, thereby increasing the water molecules’ transverse relaxation time due to pathogen-induced aggregation and disaggregation in the presence of magnetic relaxation nanosensors (MRnS).^46−48^ Viral fusion with the LIONs can be simultaneously verified via fluorescence modalities, whereby the penetration of the fusion spike into the LIONs membrane releases encapsulated DiI dye into solution.^45^ However, these studies are limited in their broader extension, as they focus narrowly on the viral fusion event itself. Thus, no studies have examined the efficacy of integrating specific receptors onto the LION platform to examine viral fusion mechanisms. To address this gap, we propose synthesizing LIONs with integrated receptors to mimic magnetic-labeled host membranes as shown in Scheme 1B. Specifically, we evaluate the feasibility of using the LIONs-ACE2 platform to measure enveloped SARS-CoV-2 viral fusion in real time by appending ACE2 receptors to the LION platform. This fusion assay takes advantage of SARS-CoV-2 spike protein activation in the presence of proteases or reduced pH, which subsequently increases the number of spike protein interactions with LIONs-ACE2 membranes. We hypothesize that as spike proteins cluster around LIONs-ACE2, they displace the surrounding water protons, thereby increasing transverse relaxation times. These changes can be quantified using a benchtop NMR instrument. Although this specific LIONs-ACE2 study investigates the fusogenic properties of SARS-CoV-2 and the ACE2 receptor, the underlying technique offers the potential to adaptively mimic a broad array of potential host cell receptors, highlighting LIONs-ACE2's flexibility and utility in quantitatively detecting fusion interactions in enveloped viruses that require a protein receptor as a trigger for membrane fusion. Thus, this approach can be extended beyond SARS-CoV-2 to rapidly screen the effectiveness of antiviral therapeutics and potential fusion inhibitors for other enveloped viruses, thereby overcoming the inherent limitations of other techniques for detecting and responding to urgent pathogenic outbreaks.

## Results and Discussion

### Synthesis and Characterization of Receptor-Conjugated Lipid-Coated Iron Oxide Nanosensors

Iron oxide (Fe_3_O_4_) nanoparticles (IONPs) were synthesized using a previously reported method and as described in the Experimental Section.^49^ Briefly, an aqueous solution of iron salt (FeCl_2_ and FeCl_3_) mixture was acid-digested before precipitating in dilute NH_4_OH solution. Polyacrylic acid (PAA) was utilized to generate thin polymer coatings around iron oxide nanocrystals and to provide excellent stability in the aqueous environment. Dynamic light scattering (DLS, Malvern’s Nano-ZS90 Zetasizer) was used for the measurement of the overall size (diameter *D* = 43.8 ± 2 nm) and surface charge (zeta potential ζ = −25.1 ± 3 mV) of the synthesized IONPs, as shown in Figure S1A,B. These results indicated the formulation of stable and dispersed IONPs. Next, a modified solvent evaporation method was used for the synthesis of LIONs, where 1,2-dioleoyl-*sn*-glycero-3-phosphocholine (DOPC) was selected as the model lipid membrane. After evaporation of the DOPC solution in chloroform, the DOPC thin film was soaked in a hydrating medium, which was a mixture of a HEPES buffer and IONP solution. Consequently, the IONPs fused into the DOPC lipid layer, which naturally formed multilamellar LIONs. An extensive extrusion process was undertaken to synthesize unilamellar LIONs. Following synthesis, the size, shape and morphology, and magnetic property of LIONs were characterized by DLS (diameter *D* = 95.2 ± 3 nm), negative stained transmission electron microscopy (TEM), and transverse relaxation as shown in Figure S2.

The synthetic protocol for LIONs was further modified to formulate metal-chelating LIONs by leveraging a previously reported technique incorporating a nickel-NTA His-tag conjugation approach^50^ as described in the Experimental Section. Synthesizing Ni^2+^-LIONs in this fashion was preferred because it was simple, no chemical modification was necessary, and the biological activity of the appended ACE2 receptor protein was maintained. Briefly, in this technique, we used a metal-ion chelating lipid composed of DOPC, cholesterol, and 1, 5, or 10 mol % of 1,2-dioleoyl-*sn*-glycero-3-{[*n*(5-amino-1-carboxypentyl)iminodiacetic acid]succinyl} (nickel salt) (DGS-NTA-Ni), which was then dissolved in chloroform, dried overnight, and hydrated with a mixture of HEPES buffer and IONPs. An extensive extrusion process was undertaken to synthesize unilamellar Ni^2+^-LIONs. These Ni^2+^-LIONs were then augmented through a Ni^2+^-NTA His-tag conjugation technique to immobilize the ACE2 receptor protein on the surface of the Ni^2+^-LIONs through noncovalent interactions.^50^ This was achieved by incubating the His-tagged ACE2 protein with Ni^2+^-LIONs for 1 h at 37 °C and then purifying the mixture with a magnetic column to remove unbound ACE2 protein. Finally, the amount of immobilized ACE2 receptor protein on the Ni^2+^-LIONs was determined by using the BCA assay to measure the amount of unbound His-tag protein remaining in the solution. LIONs lacking DGS-NTA-Ni were used to compare their binding ability toward the His-tagged ACE2 receptor protein against that of the LIONs-ACE2’s binding affinity. A similar protocol was followed for synthesizing TMPRSS2 and GM1conjugated LIONs.

**Figure 1:**
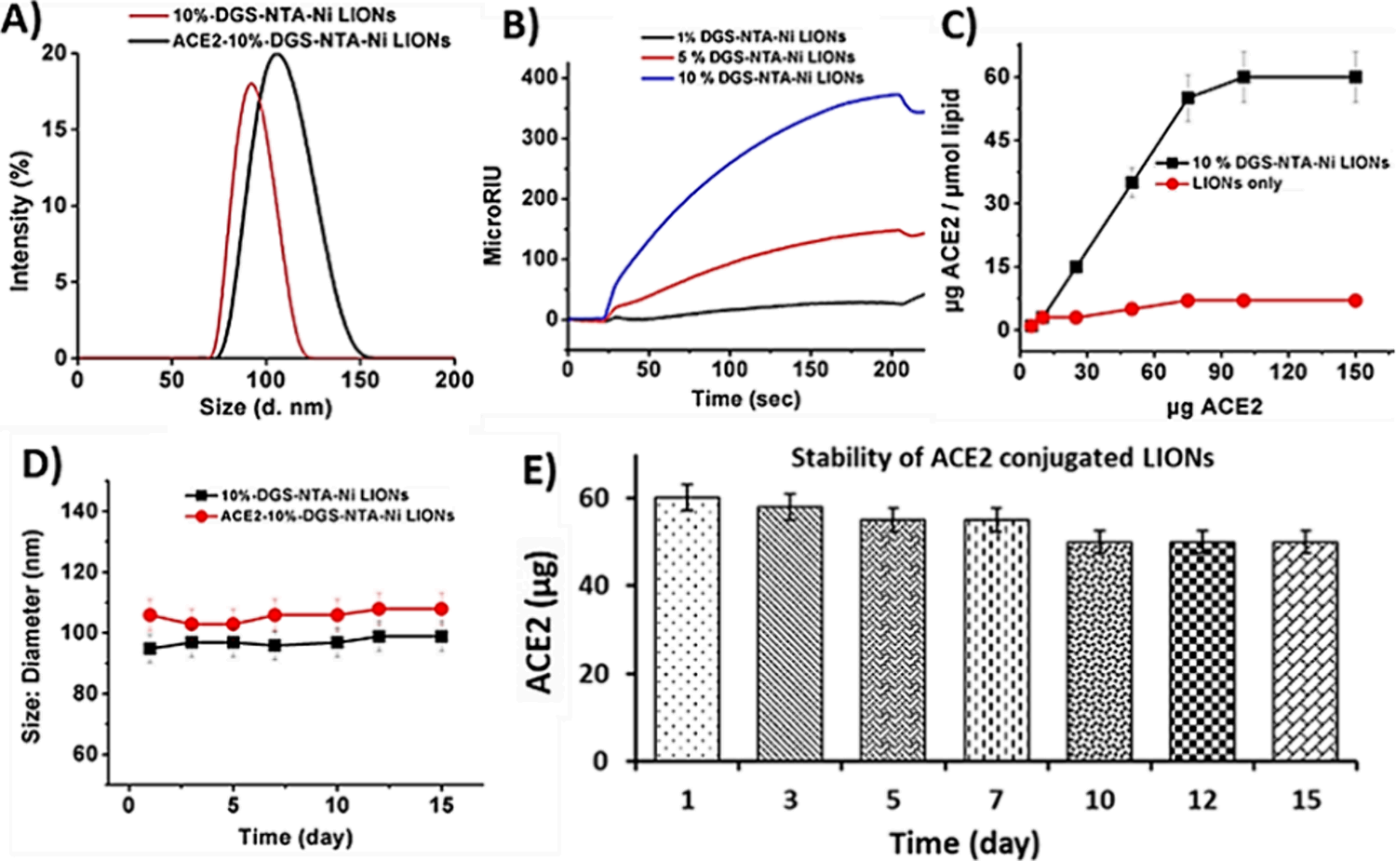
(A) Size of LIONs-NTA and LIONs-ACE2; (B) SPR of SARS-CoV-2 S protein and LIONs-ACE2 incorporating 1, 5, and 10% DGS-NTA-Ni; and (C) amount of conjugated ACE2 on 10% DGS-NTA-Ni LIONs by BCA assay. The stability of LIONs-ACE2 was evaluated by using the (D) DLS and (E) BCA assay.

Synthesized Ni^2+^-LIONs were characterized by various techniques after passing through a 0.2 μm filter to separate from any larger size particles. They were found to have an average size of 98 ± 1 nm with a mean polydispersity index of 0.1. No significant difference in size was witnessed when increasing the molar % of DGS-NTA-Ni. Next, a comparison was undertaken regarding the coupling efficiency of LIONs containing 10% DGS-NTA-Ni and His-tagged ACE2 protein at several concentrations (20–150 μL, 1.0 mg/mL). There was an increase in the hydrodynamic radii of Ni^2+^ chelating LIONs from *D* = 98 ± 1 to 105 ± 2 nm after association with ACE2 (Figure 1A). This was cross-validated by performing SPR experiments, as the highest binding response with the SARS-CoV-2 spike protein was observed in the presence of 10% DGS-NTA-Ni LIONs (Figure 1B). The mol % of DGS-NTA-Ni was not increased above 10%, as signs of aggregation were observed beyond this threshold. Moreover, as increased quantities of His-tagged ACE2 protein were added, the amount of immobilized protein on the surface of 10%-DGS-NTA-Ni LIONs also increased, ultimately plateauing when 75 μL (1.0 mg/mL) of His-tagged ACE2 protein was added, thereby exhibiting a typical BCA assay binding isotherm (Figure 1C). A control experiment was also performed with LIONs lacking DGS-NTA-Ni, indicating minimum levels of interaction with the ACE2 protein. To determine the stability of ACE2 conjugated LIONs in PBS buffer (pH 7.4), the overall size and the amount of immobilized ACE2 were measured over a period of 2 weeks using DLS (Figure 1D) and BCA protein quantification methods (Figure 1E), respectively. Minimal changes in the size were observed. Moreover, the amount of immobilized ACE2 also remained similar over time, indicating the formation of a stable conjugate. In addition, the stability of synthesized LIONs-ACE2 was evaluated over a period of several hours and at various pH levels. As shown in Figure S3, minimum variations were observed. Following a similar protocol, other receptor conjugated LIONs were formulated. As shown in Figure S4, the average sizes of TMPRSS2-LIONs and TMPRSS2-LIONs-ACE2 were found to be 107 ± 1 and 109 ± 2 nm, respectively.

### Detection of Fusion Interactions between LIONs and Spike Protein

**Figure 2:**
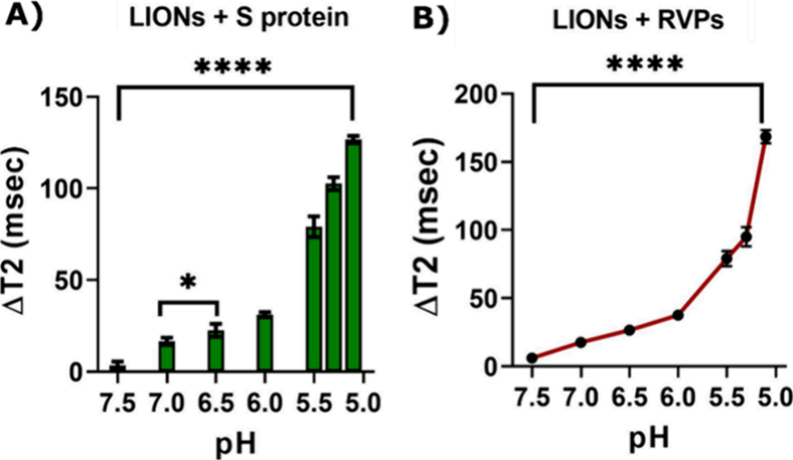
Interaction of (A) LIONs and (B) RVPs at various pH values. An increase in Δ*T*_2_ was observed following the incubation of LIONs with S/RVPs at lower pH levels.

Before performing the fusion interactions, postcoupling ACE2 enzymatic activity was determined. The enzymatic activity of the ACE2 receptor protein before and after coupling was compared using the angiotensin II converting enzyme (ACE2) activity assay kit (Abcam). In this assay, an enzymatically active ACE2 cleaved a synthetic MCA-based peptide substrate, thereby releasing a free fluorophore that could be quantified as shown in Figure S5. To obtain a baseline for comparative fusion interactions, it was first necessary to evaluate how the LION platform interacted with the SARS-CoV-2 spike protein and reporter virus particles (RVPs). The change in the transverse relaxation time was quantified at different pH levels and tested for statistical significance. Figure 2A reveals statistically significant Δ*T*_2_ changes in fusion interactions between the LIONs and SARS-CoV-2 spike protein (S) subunit between pH 7.0 (near 15 ms) and 6.5 (near 20 ms) and more significantly between pH 7.5 (near 0 ms) and 5.0 (near 150 ms). In addition, fusion interactions between LIONs and SARS-CoV-2 reporter viral particles (RVPs) that are antigenically equivalent to wild-type SARS-CoV-2 viruses were also investigated. As shown in Figure 2B, fusion interactions between LIONs and RVPs were statistically significant between pH 7.5 and 5.0, with transverse relaxation times changing from nearly 0 to roughly 175 ms, respectively.

### Evaluating the Fusogenicity of SARS-CoV-2 to LIONs Conjugated with Different Proteins

**Figure 3:**
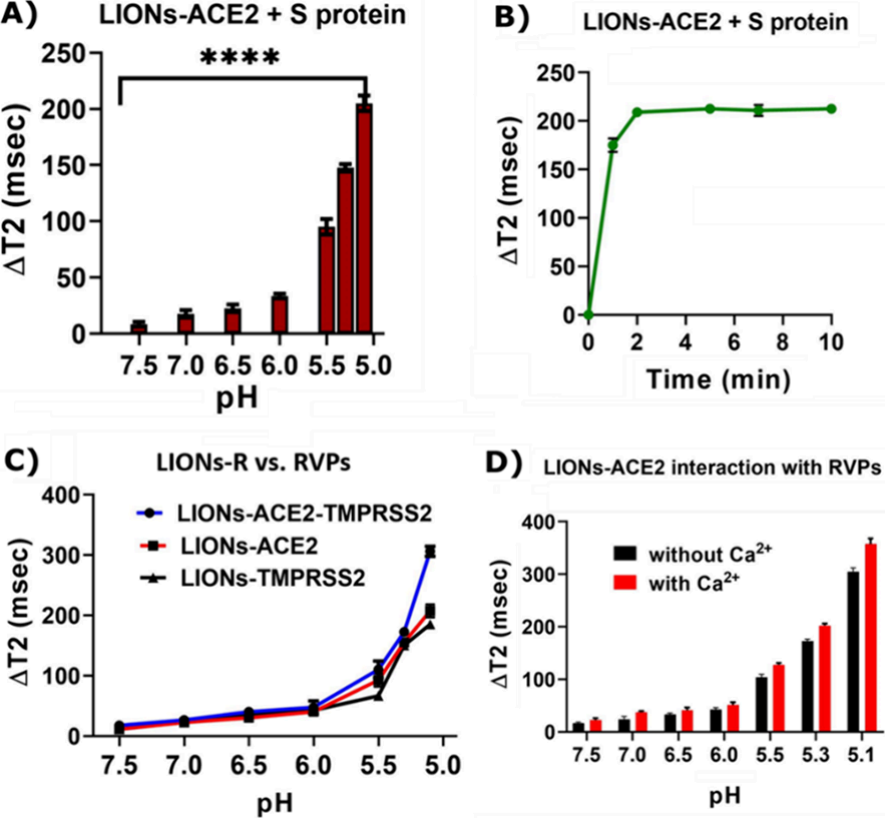
Fusion interactions between (A) LIONs conjugated with ACE2 and S protein at different pH values and (B) time-dependent interaction between LIONs-ACE2 and S protein at pH 5.1. (C) Extent of fusion interactions between RVPs and LIONs conjugated with different receptors (TMPRSS2/ACE2/TMPRSS2 + ACE2). (D) Extent of fusion interactions in the absence and presence of 0.5 mM Ca^2+^ at pH 5.1.

Next, the Δ*T*_2_ was determined for LIONs-ACE2 based on fusion interactions with the SARS-CoV-2 spike protein S subunit, and statistically significant changes were determined between pH 7.5 (near 0 ms) and 5.0 (near 200 ms) (Figure 3A). Furthermore, the Δ*T*_2_ was used to measure time-dependent fusion interactions between the LIONs-ACE2 with the SARS-CoV-2 spike protein S subunit, and results demonstrate that a stable Δ*T*_2_ near 200 ms was achieved within 2 min at pH 5.1 (Figure 3B). Interestingly, receptors other than ACE2 have also been shown to demonstrate an affinity for binding to the SARS-CoV-2 spike protein and facilitating viral entry into the cell. To compare the extent of SARS-CoV-2 fusion mechanism to LIONs conjugated with protein receptors other than ACE2, changes in Δ*T*_2_ were also used to evaluate fusion interactions between LIONs-ACE2-TMPRSS2 (near 320 ms at pH 5.0) and LIONs-TMPRSS2 (near 190 ms at pH 5.0) (Figure 3C). The fusion interaction between LIONs-ACE2 with RVPs was also evaluated in the presence and absence of 0.5 mM Ca^2+^, as prior studies have demonstrated that Ca^2+^ plays a role in the SARS-CoV-2 viral entry process by interacting with specific amino acid residues on the spike protein’s fusion peptide.^51−53^ At all measured pH gradients from 7.5 to 5.1, the Δ*T*_2_ was slightly higher in the presence of Ca^2+^, culminating in a Δ*T*_2_ near 330 ms at pH 5.1 versus a Δ*T*_2_ near 300 ms at pH 5.1 in the absence of Ca^2+^ (Figure 3D).

**Figure 4:**
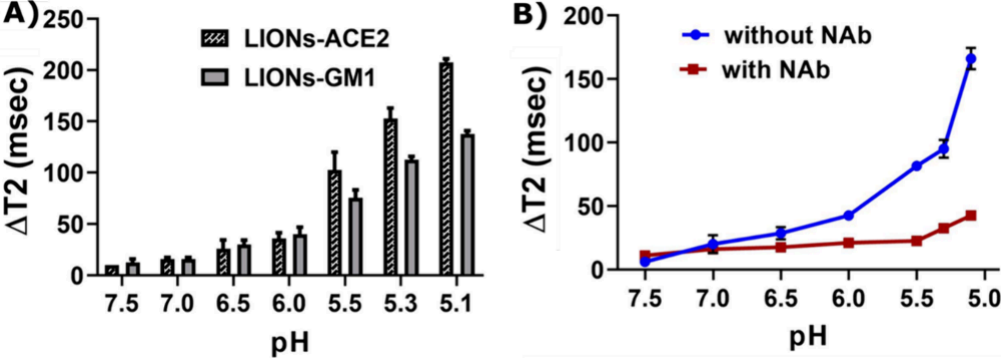
(A) Comparison of fusion interactions between RVPs and LIONs conjugated with ACE2/GM1. (B) Fusion interactions in the absence and presence of neutralizing antibody (NAb) CR3022.

In addition, other important experiments were conducted to examine the fusion interactions of LIONs-ACE2 and SARS-CoV-2 compared against LIONs-GM1, where GM1 is sialic acid containing glycolipids and known to facilitate target cell binding and fusion of influenza viruses.^2−4^ As the pH decreased in these experiments, fusion interactions between SARS-CoV-2 and LIONs-ACE2 and LIONs-GM1 both increased. At pH 5.1, the Δ*T*_2_ for LIONs-ACE2 was near 200 ms, while for LIONs-GM1, it was near 145 ms (Figure 4A). Although these differences were not statistically significant, they do offer room for further investigation to determine whether the GM1 receptor could serve as an alternate receptor utilized by SARS-CoV-2 for fusion-mediated cell entry. In fact, recent simulation studies by Mukhopadhyay and Nguyen et al. suggest that direct or indirect fusion interactions between the SARS-CoV-2 spike protein’s N-terminal domain and GM1 are possible and that sialylated glycans are able to promote viral fusion by binding to the SARS-CoV-2 spike protein RBD.^54−56^ Other researchers have posited that gangliosides like GM1 can be investigated for their potential exploitation to act as SARS-CoV-2 entry blockers.^57,58^ Finally, an experiment was conducted whereby RVPs were preincubated with the neutralizing antibody CR3022 (NAb), while control experiments were also performed with RVPs not preincubated with the neutralizing antibody. CR3022 is a cross-reactive antibody with binding affinity for both SARS-CoV and SARS-CoV-2 viruses. Upon interaction with SARS-CoV-2 in decreasing pH increments, the RVPs without the neutralizing antibody experienced far higher Δ*T*_2_ (near 165 ms at pH 5.0) compared to the RVPs with the neutralizing antibody (Figure 4B).

### Effect of Environmental Factors on SARS-CoV-2 Fusion to LIONs-ACE2

**Figure 5:**
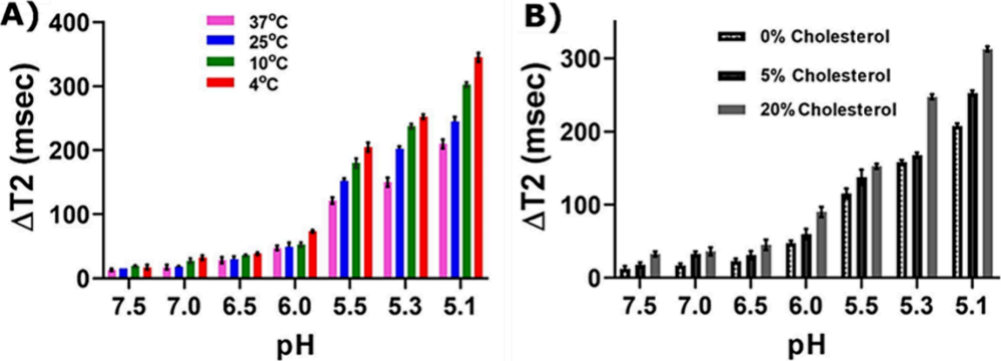
Effect of (A) temperature and (B) cholesterol compositions on fusion interactions between RVPs and LIONs-ACE2 examined at different pH values.

Experiments were conducted to evaluate how environmental factors, such as temperature and cholesterol concentration,^59,60^ affected interactions between SARS-CoV-2 and LIONs-ACE2 in terms of changes in transverse relaxation values. Before conducting the experiments to investigate the effects of different temperatures on fusion, the stability of LIONs-ACE2 at different temperatures was critically evaluated and established in an aqueous buffer over a period of 15 days by measuring the size of the formulated LIONs (Figure S6). After ensuring the stability of the LIONs-ACE2, a pH gradient from 7.5 from 5.1 was established, and certain pH levels tested the Δ*T*_2_ for SARS-CoV-2 RVPs and LIONs-ACE2 at 37, 25, 10, and 4 °C. As pH decreased, the Δ*T*_2_ values at all four temperature levels increased; however, when comparing pH 7.5 to 5.1, the most dramatic increases were at 4 °C (around 10 to 350 ms, respectively) and 10 °C (around 10 to 300 ms, respectively), as shown in Figure 5A. Less substantial Δ*T*_2_ values were witnessed at 25 and 37 °C, but even these changed dramatically from pH 7.5 to 5.1 by increasing from 10 to 250 and 10 to 200 ms, respectively (Figure 5A). Similarly, cholesterol was investigated to examine its potential role in mediating SARS-CoV-2 RVPs and LIONs-ACE2 fusion interactions. A pH gradient from 7.5 to 5.1 was established, and then 0, 5, and 20% cholesterol concentrations were analyzed. As pH decreased, Δ*T*_2_ values increased at all concentrations; however, 20% cholesterol had the largest change from pH 7.5 (20 ms) to pH 5.1 (310 ms) (Figure 5B). At 5 and 0% cholesterol, Δ*T*_2_ values also increased but to a smaller extent, with 5% cholesterol increasing from 10 to 250 ms at pH 7.5 to 5.1, while the 0% cholesterol Δ*T*_2_ values increased from roughly 10 to 200 ms at the same pH levels (Figure 5B). This study demonstrates the important role that both cholesterol and temperature play in SARS-CoV-2 fusion with host cells.

### MD Simulation Studies

**Figure 6:**
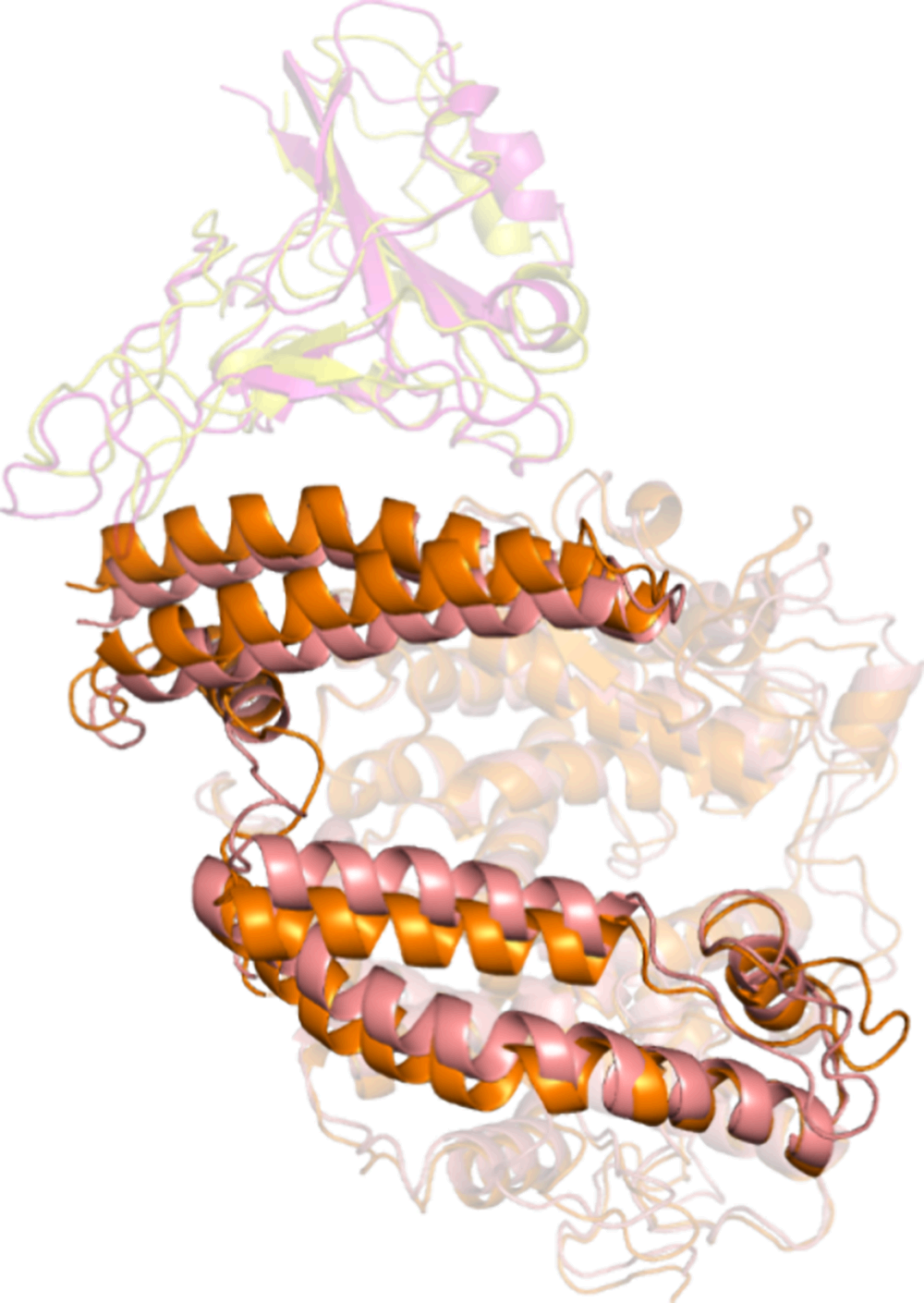
RBD-ACE2 peptidase complex orientation at 0 and 50 ns. The equilibrated complex of RBD with the ACE2 peptidase domain is shown at 0 ns (magenta and salmon) and 50 ns (orange and yellow). ACE2 residues 21–197 are shown in full opacity to highlight a hinge motion that “opens” ACE2.

The interactions between the RVPs and LIONs or LIONs-ACE2 were simplified into the most basic initial interactions for study by molecular dynamics (MD) simulation: the RBD interacting with either the peptidase domain of ACE2 or the DOPC bilayer. The spike protein can adopt opened or closed conformations, with the opened conformation exposing the RBD’s ACE2 binding site and the closed conformation burying it.^61^ Because of that, two systems with the DOPC were built with the RBD as it would be presented in either the opened or closed conformations. RBD-ACE2 simulations indicated that RBD remained bound to ACE2. Notably, with only the peptidase domain, ACE2 underwent its hinge motion that was stated as closed in the crystal structure and opened up as the simulation progressed (Figure 6). Additionally, many of the key hydrophilic interactions were maintained (Figure S7). Unexpectedly, the interaction between RBD, as it would be presented in the closed conformation of the spike protein, and the DOPC bilayer was also stable, with combined Lennard–Jones and Coulombic potentials between the RBD and the bilayer having similar interaction energies as with ACE2 (Figure S8).

**Figure 7:**
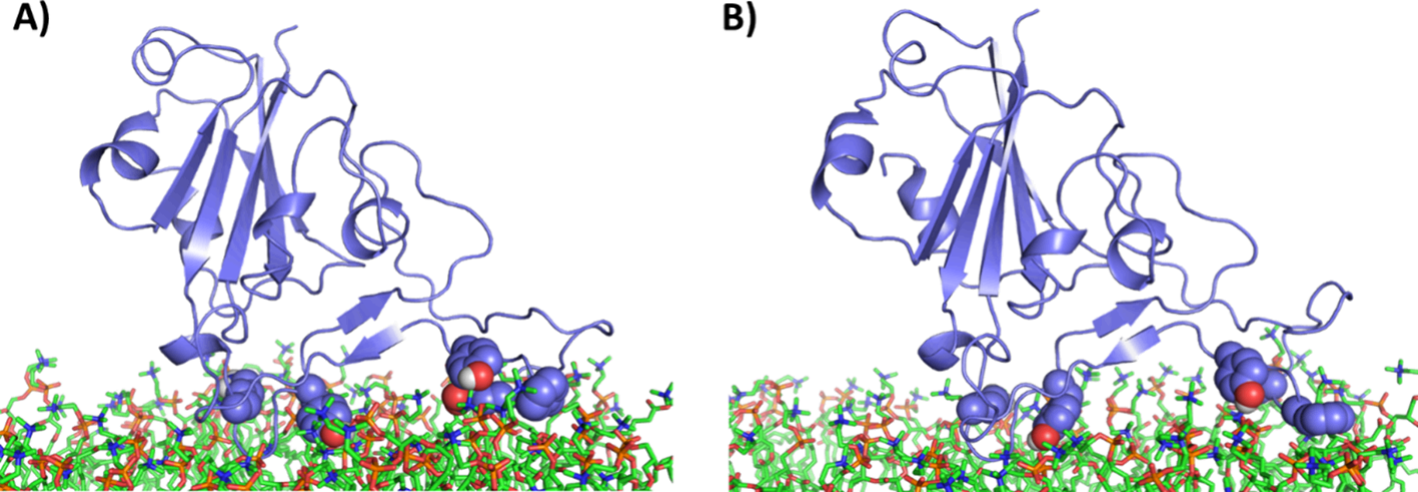
RBD-bilayer orientation at 0 and 50 ns, closed orientation. The equilibrated complex of RBD with the DOPC bilayer is shown at (A) 0 ns and (B) 50 ns. Aromatic residues that could contribute to cation-pi interactions and polar residues that could participate in salt-bridge interactions (from left to right: K444, Y449, F490, Y489, and F486) are shown as spheres.

Indeed, the visual comparison between 0 and 50 ns shows very little difference (Figure 7). Five aromatic or charged residues are positioned to make cation-pi or salt-bridge interactions with the charged phosphate or choline: K444, Y449, F490, Y489, and F486. Additionally, the side chain exposed in the closed state allows numerous hydrogen bond interactions with the DOPC head groups (Figure S9).

**Figure 8:**
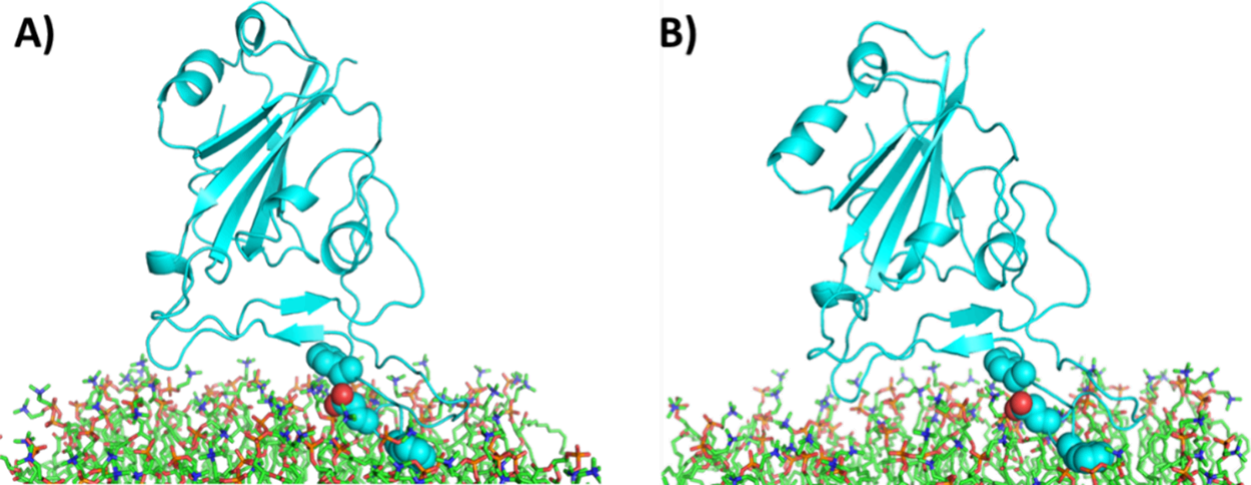
RBD-bilayer orientation at 0 and 50 ns, open orientation. The equilibrated complex of RBD with the DOPC bilayer is shown at (A) 0 ns and (B) 50 ns. Aromatic residues that could contribute to cation-pi interactions and polar residues that could participate in salt-bridge interactions (from left to right: F490, Y489, and F486) are shown as spheres.

The RBD, as it would be presented in the open conformation, was still able to have favorable interactions with the DOPC bilayer, although with a lower affinity. The interaction visually looks very similar at 0 and 50 ns (Figure 8), although there are fewer interactions available. For example, only F490, Y489, and F486 are close enough for potential cation-pi interactions with choline. Likewise, there are far fewer hydrogen bond interactions (Figure S10). The ability to bind in the open state, although with a much lower affinity, could explain the low-level background with the LIONs.

## Conclusions

As existing and emerging enveloped viruses like SARS-CoV-2, Zika, Ebola, and influenza sweep across the world and impact human health and global society, it is increasingly critical to develop techniques capable of quickly, affordably, and accurately inhibiting viral fusion mechanisms. The experiments described in this paper demonstrate the ability of our LION platform to be easily augmented into a LIONs-ACE2 platform by appending ACE2 receptors to mimic the target host cell and receptor primarily involved in SARS-CoV-2 viral fusion and pathogenesis. The data reported herein demonstrate the successful utilization of changes in transverse relaxation times to determine the fusion interactions of SARS-CoV-2 viral particles. It demonstrates the successful ability of our novel LIONs-ACE2 platform to observe viral fusion interactions using isolated viral glycoproteins and more biologically relevant models such as reporter virus particles (RVPs). In addition, we demonstrated the flexibility of the LIONs-ACE2 platform in easily mimicking other protein receptors involved in SARS-CoV-2 viral entry such as TMPRSS2 and GM1. Furthermore, the successful observation of viral fusion between the SARS-CoV-2 spike protein and the LIONs-ACE2 receptor at biologically relevant pH levels indicates that this magnetic platform may provide an adaptable avenue for analyzing viral fusion associated with many different receptors. This model also demonstrated the ability to evaluate external effects on viral fusion, including cholesterol composition, Ca^2+^ concentration, temperature, and pH level. Moreover, the use of a neutralizing antibody to prevent SARS-CoV-2 from binding to LIONs-ACE2 reveals the platform’s ability to test potential fusion inhibitors. Since understanding viral pathogenesis is a crucial step toward extrapolating techniques to inhibit viruses, the LIONs-ACE2 platform’s ability to evaluate different receptors’ binding affinities in real time highlights its candidacy to make contributions to this field.

To summarize, this article develops a novel LIONs-ACE2 platform that can be utilized to analyze the SARS-CoV-2 fusion mechanism in a rapid, customizable, and high-throughput manner using transverse relaxation as a bioanalytical tool. Significantly, this nanoplatform is relevant for broad applicability in studying manifold combinations of other enveloped viruses and protein receptors. The platform’s ability to discern fusion in real time without sophisticated equipment, while simultaneously evaluating the impacts of critical environmental factors within minutes, is a substantial contribution that could mitigate the effects of future viral outbreaks. Molecular dynamics simulations suggested that the low-level background activity observed with the LIONs lacking ACE2 could be due to nonspecific interactions with the DOPC bilayer. Finally, the robust customizability of the LIONs-ACE2 platform offers the opportunity to investigate known and emergent enveloped viruses with little to no alteration of the original LION platform.

## Experimental Section

### Surface Plasmon Resonance Experiments between DGS-NTA-Ni^2+^ LIONs and ACE2

Surface plasmon resonance (SPR, SR7500 Dual Channel System, Reichert) studies were performed to further confirm the coupling efficiency between LIONs containing 1% DGS-NTA-Ni and the His-tagged ACE2 protein. The His-tagged ACE2 was immobilized onto a CM5 sensor chip surface, which had been previously activated by EDC/NHS (40 mg/mL EDC and 10 mg/mL) coupling. Subsequently, 1 M ethanolamine (pH 8.5) was injected for 10 min to block the unreacted surfaces on the chip. Then, Ni^2+^ chelating LIONs containing differing amounts of DGS-NTA-Ni (1, 5, and 10 mol %) were flowed over the sensor chip.

### Spin–Spin T_2_ Magnetic (Transverse) Relaxation Experiments for Fusion Interaction Assays

For this experiment, we incubated 400 μL of LIONs ([Fe] = 2.0 mM) with the HEPES buffer at varying pH levels (90 μL, pH = 7.5 and 5.1) and with 10 μL of the spike protein stock solution (2 μg/mL) within a 1.0 mL relaxometer tube for 10 s. Subsequently, transverse relaxation measurements were obtained in a time-dependent manner (five times per 1 min interval) at room temperature (22 °C). A similar procedure was adopted to evaluate the effects of (1) differing cholesterol concentration in LION composition (0, 5 and 20%), (2) LIONs-ACE2 and the S1 spike protein subunit, (3) LIONs and reporter virus particles (RVPs), (4) LIONs conjugated with different protein receptors, (5) LIONs-ACE2 interactions with RVPs in the presence and absence of Ca^2+^ ions, and (6) RVPs preincubated with the neutralizing antibody CR3022 at a concentration of 4 μg/mL in the fusion interaction process.

### MD Simulation

To reduce the computational resources needed for molecular dynamics simulations, the system was decomposed into three systems: the SARS-CoV-2 spike protein RBD interacting with the ACE2 peptidase domain and the RBD interacting with a DOPC bilayer, as it would be presented in the open (ACE2-competent binding conformation) and closed (buried ACE2 interaction site) conformations. The RBD in complex with the peptidase domain of ACE2 was truncated from PDB ID 6m17,^62^ and a solvated system was built using CHARMM-GUI.^63,64^ The solvated system was a cube with a side length of 82 Å and was neutralized with two chlorine ions. The simulations were performed using GROMACS using the charmm36-jul2022 force field.^65^ A steepest decent minimization was performed followed by a 120 ps NVT equilibration at 303.15 K and by a 50 ns production NPT simulation. The two systems of the of SARS-CoV-2 RBD interacting with a DOPC lipid bilayer were also generated using the CHARMM-GUI membrane builder.^63−66^ The same RBD conformation from the ACE2 simulation was positioned and oriented as it would be when presented as either opened or closed and interacting with the lipid head groups of the bilayer. The solvated system was a rectangle with dimensions 75 × 75 × 140 Å for the open conformation and 75 × 75 × 130 Å for the closed configuration cube with a side length of 82 Å and was neutralized with two chlorine ions. The systems were minimized using steepest gradient followed by 250 ps NVT and 1625 ps NPT equilibration at 303.15 K and by a 50 ns production NPT simulation.

## Supporting Information Available

Detailed characterization data provided including dynamic light scattering, TEM, transverse magnetic relaxation, stability, and MD simulation for IONPs, LIONs, and receptor conjugated LIONs (PDF)
