# Supplementary material for: Development of Receptor-Integrated Magnetically Labeled Liposomes for Investigating SARS-CoV-2 Fusion Interactions
Source: Anal Chem. 2025 Feb 10;97(8):4490–8. doi: 10.1021/acs.analchem.4c05966 (PMC11883728; doi:10.1021/acs.analchem.4c05966)
Supplement: Supplementary file 1 — ac4c05966_si_001.pdf [file ac4c05966_si_001.pdf]

“Supporting Information”

Development of Receptor-Integrated Magnetically labeled Liposomes for Investigating SARS-CoV-2 Fusion Interactions

Tuhina Banerjee,[1,] Clayton Frazier,[1] Neelima Koti,[1] Paris Yates,[1] Elizabeth Bowie,[1] Megan Liermann,[1] David Johnson,[2] Sharon H Willis,[3] and Santimukul Santra[1,*]*

[1]Department of Chemistry and Biochemistry, Missouri State University, 901 S. National Avenue, Springfield, MO 65897, United States of America

[2]Computational Chemical Biology Core, University of Kansas, 2034 Becker Drive, Lawrence, KS 66018, United States of America

[3]Integral Molecular Incorporation, One uCity Square 25 N. 38th Street, Suite 800, Philadelphia, PA 19104, United States of America

*Corresponding authors: Santimukul Santra, Email: ssantra@missouristate.edu
Tuhina Banerjee, Email: tbanerjee@missouristate.edu

TABLE OF CONTENT

Experimental Section……………………………………………………………………S2
Characterization of LIONs………………………………………………………………S4
Stability study of LIONS-ACE2…………………………………………………………S5
DLS measuremeny of other receptor-LIONs……………………………………………S6
Enzymatic activity of ACE2……………………………………………………………..S6
DLS study of LIONs-ACE2……………………………………………………………..S7
MD simulation studies…………………………………………………………………..S7

EXPERIMENTAL SECTION

Materials and Instruments. Croda International provided 1,2-Dioleoyl-*sn*-glycero-3-phosphocholine (DOPC) and ovine cholesterol (ovine wool, >98%), and recombinant SARS-CoV-2 spike protein (ab69741) was obtained from Abcam. Anti-SARS-CoV-2 monoclonal antibody and recombinant human ACE2 protein with an His-tag were obtained from SinoBiological. Polyacrylic acid (PAA) was obtained from Sigma-Aldrich. From Acros Organic, chloroform (99.8+%) and HEPES sodium salt (99%) were procured. Chemicals including ammonium hydroxide (NH_4OH), hydrochloric acid (HCl), ferrous chloride tetrahydrate, and ferrous chloride hexahydrate ($FeCl_2·4H_2O$, $FeCl_3·6H_2O$) were purchased from Fischer Scientific. Sigma Aldrich provided transmembrane serine protease 2 (TMPRSS2) and monosialotetrahexosylganglioside (GM1), while SARS-CoV-2 Reporter Virus Particles (RVPs) were a kind gift from Integral Molecular. Magnetic relaxometer mq20 (0.47 T) from Bruker was utilized for transverse relaxation measurements, while a Zetasizer-ZS90 from Malvern allowed for the determination of IONP surface charge and size characterizations. Transmission electron microscopy (TEM) experiments were conducted using JEOL-JEM 2100 electron microscopes. Croda International also provided an extruder system that was utilized for the LION synthesis. Miltenyi Biotech provided a magnetic column setup. Finally, an ACE2 activity assay kit was obtained from Abcam.

Synthesis of polyacrylic acid iron oxide nanoparticles (PAA-IONPs). Iron oxide nanoparticles were prepared using our previously reported protocol.[49] During our synthetic approach, a core of iron oxide (Fe_3O_4) was initially generated, which was subsequently layered with a coating of polyacrylic acid. Three initial solutions were created: Solution 1 consisted of polyacrylic acid (0.859 g) dissolved in water (5 mL); Solution 2 consisted of NH_4OH (1.8 mL from a 30% stock solution) and was further diluted with 15 mL of water; Solution 3 contained a mixture of $FeCl_2·4H_2O$ (0.334 g) and $FeCl_3·6H_2O$ (0.622 g) dissolved in water (2 mL). After these solutions were prepared, HCl (90 µL from 12 M stock) was added to Solution 3, and then Solution 2 was immediately added to Solution 3 while mixing. Then, Solution 1 was added to this solution, and the resulting mixture was vortexed for roughly one hour at 3000 rpm. This solution was subsequently centrifuged at 4000 rpm for 30 minutes to eliminate nanoparticle agglomerates and large nanoparticles. The resulting solution was purified using a QuadroMACS™ LS magnetic column and then by dialysis (MWCO 6-8 KDa) in PBS (pH= 7.40). The iron concentration was determined by evaluating acid digestion. The nanoparticles were subjected to acid digestion using HCl, which converts all Fe ions to iron (III). The amount of iron was estimated by generating a standard calibration curve of known concentrations of $FeCl_3$, and the absorbance was subsequently recorded at 410 nm after the acid digestion. These IONPs ([Fe] = 5 mM) were then characterized in terms of their size and surface charge using DLS, and confirmation of a successful PAA polymer coating was verified by measuring a negative zeta potential.

Synthesis of liposome-coated iron oxide nanoparticles (LIONs). Liposome-coated iron oxide nanoparticles were synthesized using our previously reported protocol.[45] In short, a solution was first prepared by dissolving DOPC (100 mg) in chloroform (1 mL). Next, a dry lipid film was created by evaporating the chloroform solvent under reduced pressure (600 mmHg) using a vacuum pump for 6 h. This layer was then hydrated using a stock solution of IONPs (4 mL, 5 mM) and HEPES buffer (4 mL, pH = 7.40). This hydrated layer was mixed at room temperature (22 °C) for 3 h, and the resulting solution was centrifuged at 7000 rpm for 25 min to eliminate any unbound DOPC. Next, the solution was extruded through a 100 nm polycarbonate membrane at least 21 times to decrease polydispersity and to convert the multilamellar vesicles into unilamellar liposomes. The iron concentration [Fe] of synthesized LIONs was found to be 5.0 mM. Finally, non-entrapped, excess IONPs were removed using a PD-10 column and through final purification via a magnetic column, thereby removing free lipids.

Synthesis of nickel-ion chelating LIONs (LIONs-Ni^{2+}). To synthesize LIONs-ACE2, we utilized a nickel-NTA His-tag conjugation approach.[49] A metal-ion chelating lipid was initially utilized to generate LIONs. To begin, DOPC, cholesterol, and 1,2-dioleoyl-*sn*-glycero-3-{[*n*(5-amino-1-carboxypentyl)iminodiacetic acid]succinyl} (Nickel Salt) (DGS-NTA-Ni) were purchased from Croda International, and subsequently dissolved in chloroform. Next, this solution was dried overnight and subjected to hydration with PAA-IONPs (3 mM) in a 10 mM HEPES buffer (pH 7.4). After mixing, the resulting multilamellar vesicles (MLV) were extruded for 21 cycles to form unilamellar nickel-LIONs. During optimization, the applied concentrations of DGS-NTA-Ni (1, 5, 10 mol %) were varied. LIONs lacking DGS-NTA-Ni altogether were also synthesized, and their binding affinity toward the SARS-CoV-2 spike protein was compared against the binding affinity of His-tagged ACE2 LIONs. The DGS NTA-Ni lipid randomly dispersed its polar head groups along the outer LIONs surface and was therefore a participant in non-covalent interactions with the His-tagged ACE2 protein.

Coupling of His-tagged ACE2 with LIONs-Ni^{2+}. The previously mentioned Ni^{2+}-NTA His-tag conjugation approach was employed to immobilize ACE2 receptor proteins onto the surface of Ni^{2+} chelating LIONs. DLS and TEM were subsequently utilized to characterize the Ni^{2+} chelating LIONs both before and after coupling with His-tagged ACE2. A hydrodynamic radius and a polydispersity index for the LIONs-ACE2 were established to assess their uniformity and size before and after conjugation. This approach was likewise utilized to generate additional functional LIONs by immobilizing TMPRSS2, ACE2-TMPRSS2, and GM1 receptor proteins onto the surface of LIONs.

Stability of LIONs and LIONs-ACE2 at Different pH. The stabilities of LIONs-ACE2 at different pH were evaluated utilizing a dynamic light scattering approach. First, the stock LIONs solution was diluted to [Fe] = 2.0 mM by adding HEPES buffer (pH 7.5). During the stability studies, a 400 μL solution of LIONs ([Fe] = 2.0 mM) was mixed with 100 μL of HEPES buffer solutions of varying pH (1X; pH: 7.5, 7.0, 6.5, 6.0, 5.5, 5.3, 5.1), and the various sizes of LIONs-ACE2 solutions at different pH levels were monitored using dynamic light scattering every 24 h. The HEPES buffer solutions (1X) with varying pH levels were previously prepared by adding aliquots of 50 mM sodium citrate (pH 3.0) to HEPES buffer containing a starting pH = 7.5.

Preparation of LIONs and Spike Protein Stock Solutions for Fusion Protein–Membrane Interaction Assays. To prepare for the fusion assay, the concentration of synthesized LION solution ([Fe] = 5.0 mM) was diluted by adding HEPES buffer (pH 7.5), and the final concentration was adjusted to [Fe] = 2.0 mM. This yielded a baseline transverse relaxation value of 110 ms (**Figure S2C**). This LIONs stock solution was utilized for all LION-based and LIONs-receptor-based fusion experiments. The stock solution of viral SARS-CoV-2 spike protein was prepared by adding 1 μL (1.3 μg/μL) of spike protein to 999 μL of deionized water (pH 7.5) to generate a final stock concentration of 1.3 μg/mL.

Transmission Electron Microscopy (TEM) Imaging of LIONs by Negative Staining. Transmission electron microscopy experiments were conducted on JEOL-JEM 2100 to determine the size and morphology of LIONs. Briefly 5 μL of LION solution was applied on copper grid (Electron microscopy Sciences, EMS) for one minute and were allowed to dry in the air before the sample was placed on the microscope.

CHARACTERIZATION OF LIONs:

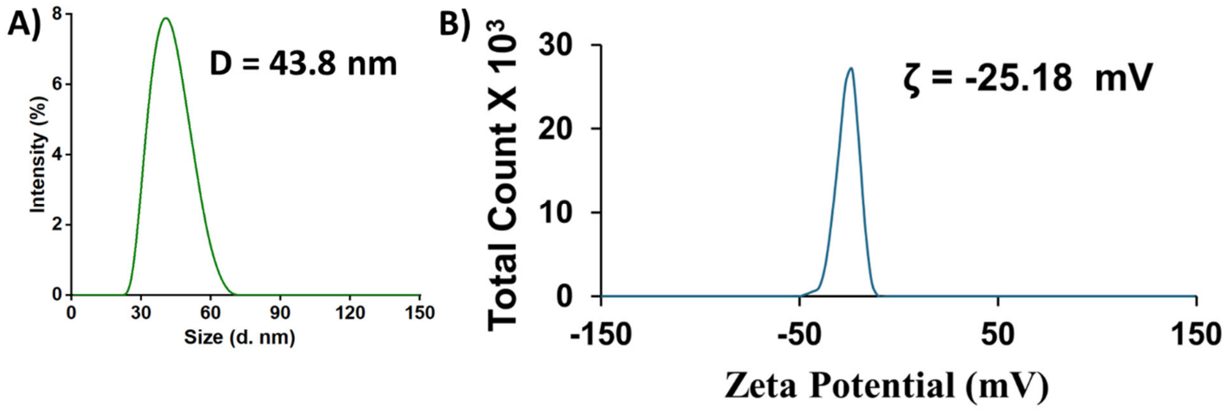

Figure S1: Determination of (**A**) hydrodynamic diameter and (**B**) zeta potential of the PAA-coated IONPs using DLS.

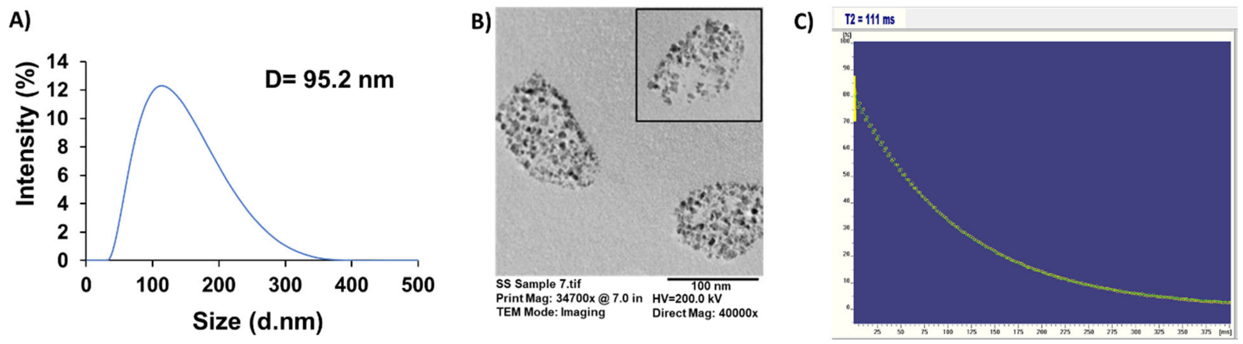

Figure S2: Size, morphology and magnetic relaxation determination of LIONs using (**A**) DLS, (**B**) TEM, Inset: image with a lesser background noise (higher contrast), and (**C**) spin-spin T_2 magnetic relaxation measurements.

STABILITY STUDY OF LIONs-ACE2:

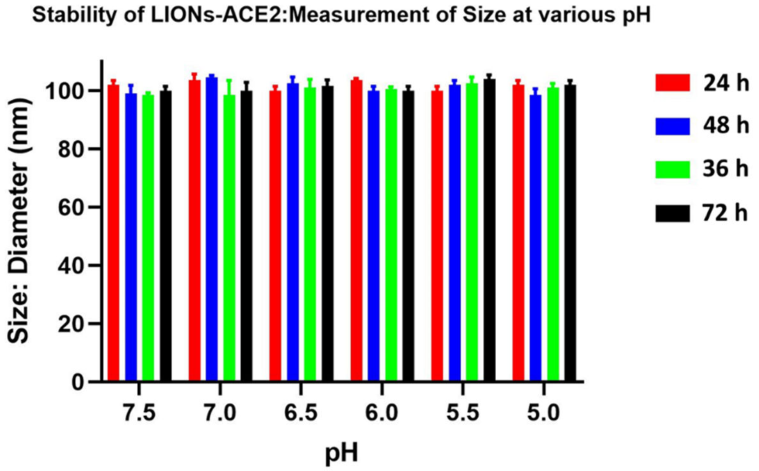

Figure S3: Assessment of stability by determining the size of LIONs-ACE2 at several pH and at different time points.

DLS MEASUREMENY OF OTHER RECEPTOR-LIONs:

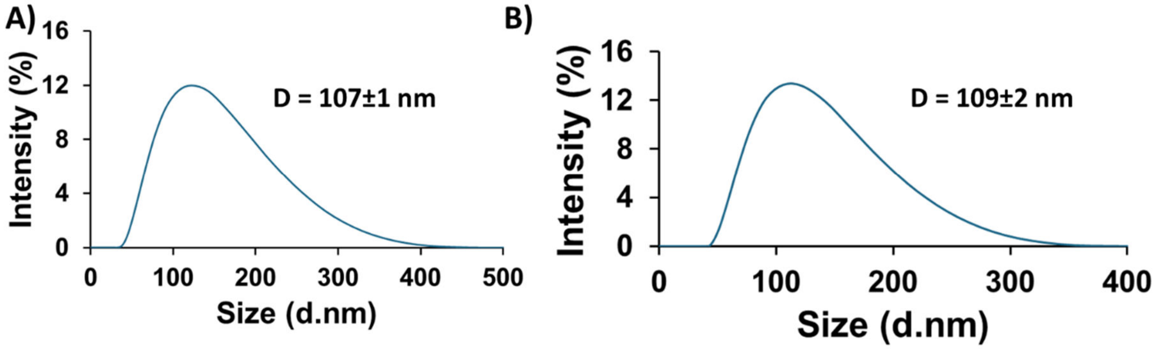

Figure S4: Determination of hydrodynamic diameters of (**A**) TMPRSS2-LIONs, and (**B**) TMPRSS2-LIONs-ACE2 using DLS.

ENZYMATIC ACTIVITY OF ACE2:

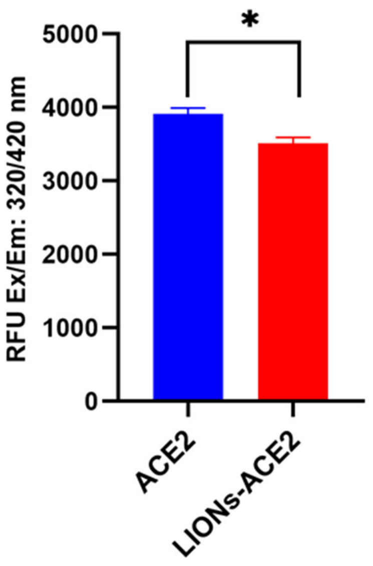

Figure S5: ACE2 activity before and after conjugating with LIONs. Data represents mean ± SEM, where n = 3 replicates. Statistical data analysis was conducted using unpaired-t test.

DLS STUDY OF LIONs-ACE2:

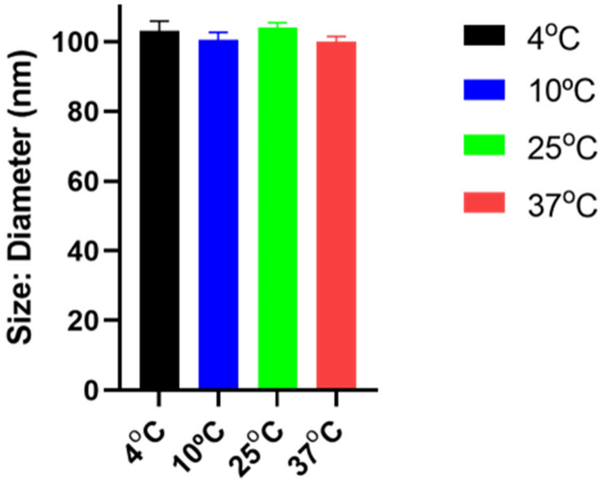

Figure S6: Determination of the size of LIONs-ACE2 at various temperature.

MD SIMULATION STUDIES:

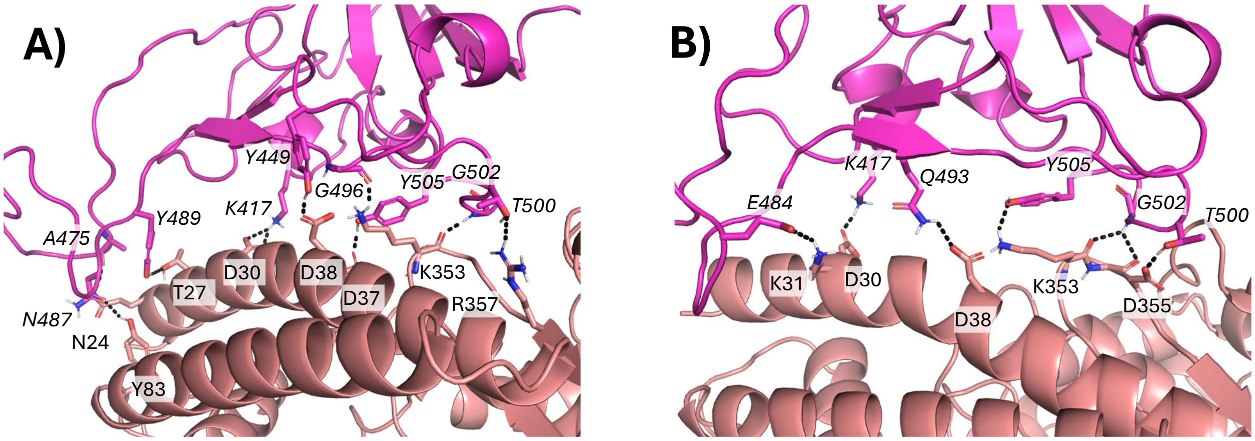

Figure S7: RBD-ACE2 peptidase complex at 0 ns, and 50 ns. The equilibrated complex of RBD (magenta) with the ACE2 peptidase domain (salmon) is shown at **A)** 0 ns, and **B)** 50 ns. Polar contacts are shown as black dashes, with residues labeled (RBD residues italicized).

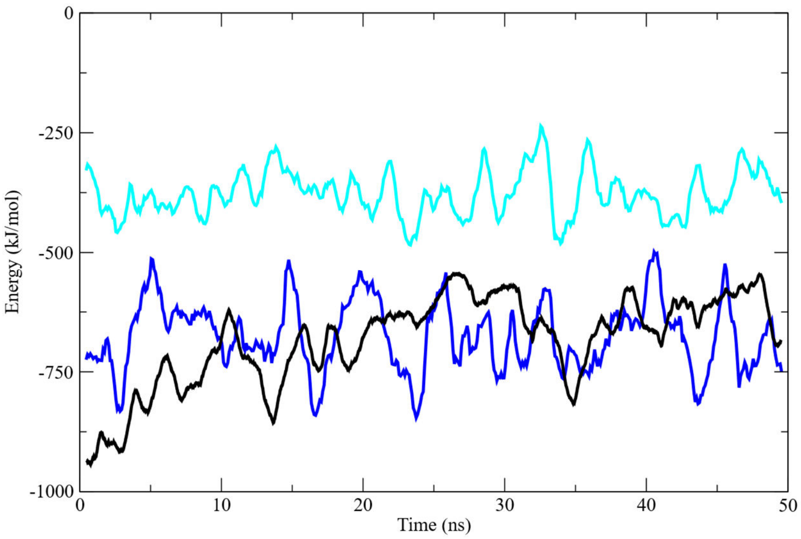

Figure S8: RBD interaction energies throughout the MD simulations. The 1 ns running average of the sum of the Lennard-Jones and Coulombic potentials for the interactions of RBD with either ACE2 (black), or the DOPC bilayer in the opened (cyan) or closed (blue) orientation.

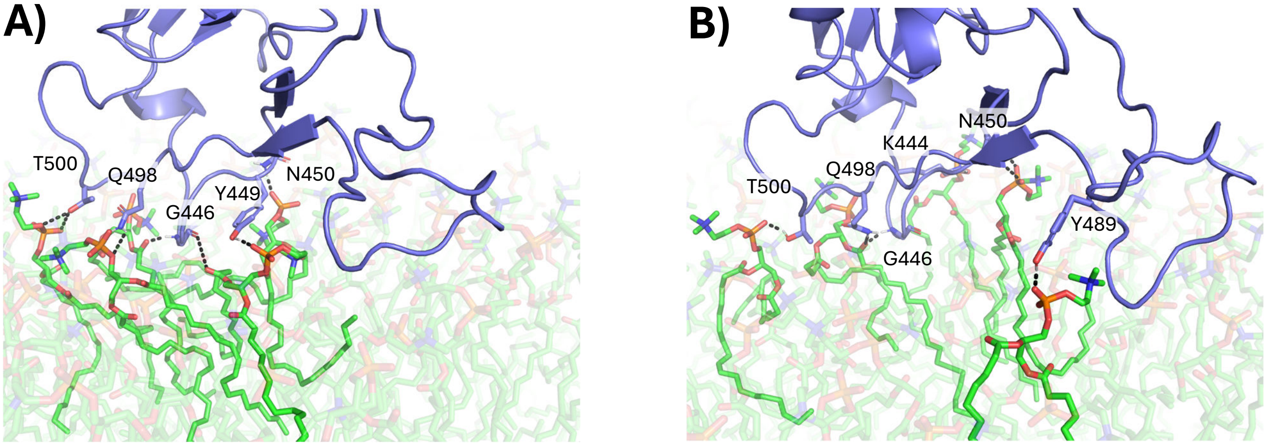

Figure S9: RBD-bilayer interactions at 0 ns and 50 ns, closed orientation. The equilibrated complex of RBD with the DOPC bilayer is shown at **A)** 0 ns, and **B)** 50 ns. Polar contacts are shown as black dashes, with RBD residues labeled.

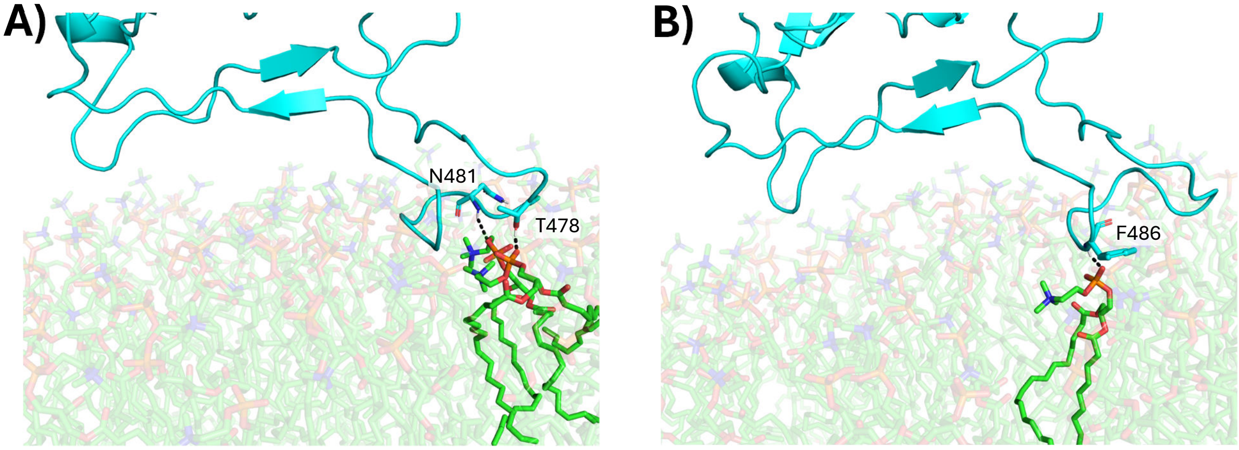

Figure S10: RBD-bilayer interactions at 0 ns and 50 ns, open orientation. The equilibrated complex of RBD with the DOPC bilayer is shown at **A)** 0 ns, and **B)** 50 ns. Polar contacts are shown as black dashes, with RBD residues labeled.
